# Predicting the next move: tracking the complexity of lung cancer evolution and metastasis

**DOI:** 10.1038/s41392-023-01567-5

**Published:** 2023-08-09

**Authors:** Carina Lorenz, Axel M. Hillmer, Johannes Brägelmann

**Affiliations:** 1 University of Cologne, Faculty of Medicine and University Hospital Cologne, Department of Translational Genomics, Cologne, Germany; 2 University of Cologne, Faculty of Medicine and University Hospital Cologne, Mildred Scheel School of Oncology Cologne, Cologne, Germany; 3 University of Cologne, Faculty of Medicine and University Hospital Cologne, Center for Molecular Medicine Cologne, Cologne, Germany; 4 University of Cologne, Faculty of Medicine and University Hospital Cologne, Institute of Pathology, Cologne, Germany

**Keywords:** Cancer genomics, Lung cancer, Genome informatics

In a series of seven papers in Nature and Nature Medicine, the TRACERx study group presents multi-omic analyses of 421 non-small cell lung cancer (NSCLC) patients, which have yielded a comprehensive map of intratumor heterogeneity (ITH) and lung cancer evolutionary trajectories at an unprecedented level of detail.^1–7^ The in-depth molecular data, and their subsequent integrated analyses, hold the potential to not only shine light on lung cancer progression but also influence a broad spectrum of scientific disciplines, research fields, and clinical oncology, thereby enhancing our understanding of cancer in general.

**Fig. 1:**
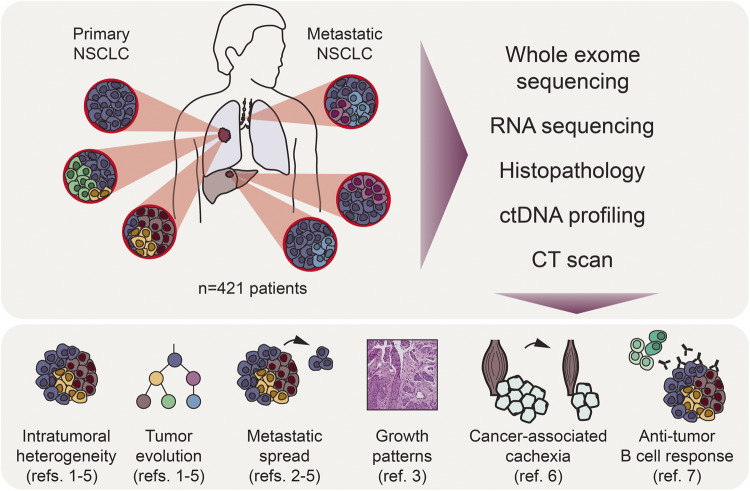
Study workflow for first *n* = 421 TRACERx Non-Small Cell Lung Cancer (NSCLC) patients published in.^1–7^ Samples and patients were primarily analysed by multiple modalities including sequencing of DNA, RNA or circulating tumor DNA (ctDNA) and/or Computed Tomography (CT) scans (right). Results were integrated with clinical information and additional data sources to study six main topics (bottom)

For their investigations, TRACERx builds on the advancements in sequencing technologies that over the last two decades transformed our understanding of cancer genomics and tumour biology, including NSCLC. Those advancements moreover enabled precision medicine, which significantly improved NSCLC patient survival. However, intratumoral heterogeneity (ITH) and genetic shifts during a tumour’s life history and progression still present major challenges to treatment.^8^ However, studying these phenomena requires sampling multiple time-points and multiple body sites (e.g. tumour and metastases), a particularly difficult task for lung cancers. TRAcking non-small cell lung Cancer Evolution through therapy (TRACERx), a large-scale study headed by Charles Swanton in the UK, set out to understand the evolutionary trajectories and their clinical consequences in NSCLC. The recent papers now published findings of the first 421 prospectively recruited patients with early-stage NSCLC. To characterise ITH, tumour evolution and its relation to clinical outcome, tumours were analysed in a multimodal approach utilising genomic, transcriptional, and histological profiling. Each method was applied independently to multiple tumour regions per sample to fine-map ITH and -where available- to primary tumours, metastases and relapsed tumours. They also studied circulating tumour DNA and body composition to investigate the relationship between molecular alterations and cachexia and investigated the role of B cells in anti-tumour immunity (Fig. 1).

Consistent with previous findings,^9^ whole-exome sequencing of 1644 regions from 421 primary tumours showed that high ITH of somatic copy number alterations but not of point mutations was associated with shorter disease-free survival.^1^ Similarly, subclonal whole genome doubling was significantly associated with shorter DFS while clonal whole genome doubling did not show such an association.^1^ Assessment of transcriptomes identified a set of highly expressed cancer genes with minimal transcriptional ITH, which also showed genetic evidence of being positively selected during tumorigenesis. Comparison of selection signals between truncal and subclonal mutations in cancer genes allowed mapping the role of these genes to early or late tumour evolution. Significant subclonal but not truncal alterations affected known driver genes such as *PTEN* in lung adenocarcinoma, indicating a role in late rather than early tumour evolution.^2^ Positive selection of subclonal mutations was observed to be stronger in cases of clonal illusion, i.e. one subclone dominating a tumour region and evidence of recent subclonal expansion. Phenotypically, tumours with recent subclonal expansion were highly proliferative and displayed high-grade growth patterns histologically.^2,3^ Interestingly, recent subclonal expansion was significantly associated with shorter disease-free survival,^1^ suggesting that subclonal dynamics are major determinants of clinical trajectories.

To identify evolutionary patterns associated with progression, 126 patients with paired primary tumours and lymph node metastasis tissue in the TRACERx cohort were identified. Among primaries and metastases, the majority (68.9%) of driver mutations were shared, whereas 75% of metastases diverged late, i.e. after the last subclonal expansion (clonal sweep) in the primary tumour. In case of early divergence, simulations suggested that these metastases likely derive of tumours smaller than 8 mm in diameter, a typical size threshold in computed tomography to trigger further investigation. Overall, metastases are more likely seeded from the primary tumour than from existing metastatic sites. Comparing seeding and non-seeding clones of the primary tumour revealed distinct genomic and phenotypic characteristics like mutations in *NRAS*, *TP53* or *RBM5*, recent subclone expansion or a highly proliferative phenotype.^4^ While most metastases are seeded by a single clone, polyclonal seeding is an additional risk factor associated with extra-thoracic disease recurrence and poor clinical outcome.^4^ The true rate of polyclonal seeding however is challenging to judge owing to potential undersampling of metastases e.g. due to inaccessible metastatic location. To mitigate this limitation, the TRACERx group developed a novel approach to derive subclonal structure of recurring tumors using circulating tumour DNA (ctDNA).^5^ Analysis of ctDNA could furthermore be utilised to detect cases of polyclonal seeding. Matched pre- and postoperative profiling ctDNA revealed that subclones which are detected postoperatively are already expanded before surgery, as indicated by higher preoperative cancer cell fractions. Since the postoperatively detectable subclones are reflective of recurrent disease, the preoperative cancer cell fraction might inform about the metastatic potential of a specific clone and can be utilised to predict metastasis.^5^

Expanding beyond tumour evolution, the TRACERx team used their molecular data to predict the onset of cachexia, a severe, frequently seen complication of advanced tumours marked by fat and muscle loss.^6^ CT scans at surgery and again at disease relapse were used to identify patients that developed cachexia and those that did not. Analysis revealed that primary tumours of cachexia patients had increased inflammatory and epithelial-mesenchymal transition signalling as well as genomic alterations in known cachexia candidate genes. Plasma proteome profiling suggested GDF15 as a potential circulating mediator of cachexia.^6^ While the direction of causality remains to be determined, these molecular profiles could help to identify patients with high risk of cachexia.

Furthermore, TRACERx researchers explored the relationship between cancer treatment and anti-tumour immune response.^7^ In mouse models and patient samples, they discovered B-cell proliferation in tumour-adjacent tertiary lymphoid structures (TLS) that were increased by immunotherapy and KRAS inhibition. Presence of TLSs has been correlated with improved immunotherapy outcomes previously, but the reasons were unclear. Surprisingly, the B-cell antibodies did not target tumour-specific neoantigens, but instead bound envelope proteins of endogenous retroviruses (ERVs), which are retroviral sequences embedded in human DNA that can reactivate in cancer cells. ERV-reactive antibodies prolonged survival in mice and correlated with immunotherapy-response in lung cancer patients, potentially providing a novel mechanistic link between TLSs and immunotherapy response.

Overall, the multi-omic analysis of this prospective, extensively sampled NSCLC patient cohort provides unparalleled insights into the complexity of tumour architecture and evolution. It emphasizes the vital relevance of clonal dynamics regarding disease progression and underscores the need for ongoing disease assessment to refine treatment approaches. Simultaneously, TRACERx pioneers new strategies, such as utilizing molecular data for cachexia prediction or inferring tumour evolution and clonality from ctDNA. It thereby provides a rich resource of data, methods and hypotheses for follow-up analyses and experimental studies investigating mechanistic connections between genetics, evolutionary dynamics and disease progression.
